# Connecticut Valley Dental Association

**Published:** 1865-12

**Authors:** 


### Proceedings of Societies.

### CONNECTICUT VALLEY DENTAL ASSOCIATION.

The Annual meeting was held in Springfield, Mass., Oct. 31st. Over forty members were present.

The following were elected officers for the ensuing year: President, J. Beals, Greenfield, Mass.; Vice-Presidents, J. McManus, Hartford, Conn., and O. F. Harris, Worcester, Mass.; Secretary, L. D. Shepard, Salem, Mass.; Treasurer, C. S. Hurlbut, Springfield, Mass.; Executive Com., E. E. Crofoot, Hartford, Conn., F. C. Buckland, Manchester, Conn., Ralph Morgan, Chicopee, Mass.

The retiring President, Dr. O. R. Post, of Brattleboro, Vt., read an interesting paper. Papers were read by Drs. McManus, of Hartford and Shepard of Salem.

Drs. Mills, of Brooklyn and S. P. Miller, of Worcester, were elected honorary members.

The time was devoted to the discussion of means of controlling the flow of saliva; adhesive fillings; taking impressions, and clinics, by Drs. Mills and Shepard.

A new gas inhaler was exhibited by the inventor, Dr. Bullock, of Cambridgeport, Mass., and some castings for cleft palate, with remarks theron, by Dr. Welton, of Cheshire, Conn.

After adjournment we were bountifully entertained by the Springfield friends.

The next meeting will be held in Worcester, Mass., on the 1st Tuesday in June, 1866.

